# A Web-Based Alcohol Risk Communication Tool: Development and Pretesting Study

**DOI:** 10.2196/13224

**Published:** 2020-01-02

**Authors:** Bridget Kool, Rosie Dobson, Sarah Sharpe, Gayl Humphrey, Robyn Whittaker, Shanthi Ameratunga

**Affiliations:** 1 School of Population Health Faculty of Medical and Health Sciences University of Auckland Auckland New Zealand; 2 National Institute for Health Innovation School of Population Health, Faculty of Medical and Health Sciences University of Auckland Auckland New Zealand; 3 Counties Manukau Health Auckland New Zealand; 4 Section of Epidemiology and Biostatistics, School of Population Health Faculty of Medical and Health Sciences University of Auckland Auckland New Zealand

**Keywords:** alcohol drinking, risk assessment, risk communication, harm minimization, primary care

## Abstract

**Background:**

Alcohol use is a major public health concern associated with an increased risk of morbidity and mortality. Health professionals in primary care commonly see patients with a range of alcohol-related risks and problems, providing an ideal opportunity for screening and brief intervention (BI).

**Objective:**

This study aimed to develop a prototype for a Web-based tool for use by primary care health professionals (eg, doctors and nurses) to communicate alcohol harm risk to their patients and to engage with them regarding ways this risk could be reduced.

**Methods:**

Following conceptualization and development of prototype wireframes, formative work and pretesting were undertaken. For the formative work, focus groups and key informant interviews were conducted with potential end users of the risk communication tool, including health professionals and consumers. The focus groups and interviews explored perceptions of alcohol risk communication and obtained feedback on the initial prototype. For pretesting, participants (primary care doctors and nurses) completed a Web-based survey followed by a period of pretesting before completion of a follow-up survey. The study was designed to gain feedback on the tool’s performance in real-world settings as well as its relevance, ease of use, and any suggested refinements.

**Results:**

In the formative work stage, 11 key informants and 7 consumers participated in either focus groups or individual interviews. Participants were very positive about the prototype and believed that it would be useful and acceptable in practice. Key informants identified that the key point of difference with the tool was that it provided *all the pieces* in 1 place (ie, assessment, interpretation, and resources to support change). Participants provided feedback on how the tool could be improved, and these suggestions were incorporated into the prototype where possible. In the pretesting stage, 7 people (5 doctors and 2 primary care nurses) completed the pretesting. Participants reported that the tool provided a useful framework for an intervention, that it would be acceptable to patients, that it was easy to use, that they would be likely to use it in practice, and that there were no technical issues.

**Conclusions:**

The alcohol risk communication tool was found to be acceptable and has the potential to increase the confidence of health professionals in assessing risk and providing BI.

## Introduction

### Background

Alcohol use is a major public health concern associated with an increased risk of morbidity and mortality [1]. Approximately 1 in 5 New Zealanders aged older than 15 years drinks in a way that is hazardous to their health [2]. Alcohol-related harm has an enormous impact on the lives and health of New Zealanders [3].

Alcohol screening tools are designed to help practitioners identify people not seeking treatment for alcohol problems but whose alcohol use may be harmful or people who may be at risk of an alcohol use disorder. Assessing alcohol consumption, drinking behaviors, and alcohol-related problems is relatively easy using validated clinical alcohol risk assessment tools such as the Alcohol Use Disorders Identification Test (AUDIT) [4]. However, many practitioners can find it difficult to explain to patients their risk of alcohol-related harm and how small changes can have positive benefits [5-7].

Alcohol brief interventions (BIs) are broadly defined as a single short session of structured advice and information for those identified via screening as hazardous or harmful drinkers. They can be conducted by health or social care professionals and delivered by face-to-face sessions, written self-help materials, telephone counseling, or digital programs [8,9]. Most types of alcohol screening and BI are underpinned to some degree by the *stages of change* theory [10] and motivational interviewing [11].

The efficacy and cost-effectiveness of alcohol BIs in primary care settings are well established [8,12-15]. However, primary care offers a different experience than the commonly studied emergency department or hospital setting. Primary care clinicians commonly examine patients with a range of alcohol-related risks and problems [16]. Despite this evidence, the implementation of alcohol screening and BI in primary care remains a challenge. Clinicians in primary care often lack confidence in their ability to do this and are reluctant to initiate discussions about alcohol. This reluctance is not only attributed to perceived lack of resources and training but is also compounded by heavy staff workloads and alcohol-related stigma, along with uncertainty on how to assist patients with more severe alcohol problems [6,17].

As more consumers turn to the internet for health-related information, the internet is increasingly being used by health organizations as a medium to deliver interventions, including alcohol BIs, resources, and services [18]. Web-based interventions have perceived advantages over the more traditional modes of health information delivery, in terms of acceptability and accessibility, privacy and anonymity, and the ability to reach a large audience in a cost-effective manner [19]. A number of recent systematic reviews and meta-analyses lend support to the notion that Web-based interventions offer promise as a strategy to reduce alcohol-related harm in a way that is appealing and nonintrusive to particular groups (eg, those who are unaware of their hazardous drinking behavior or those who are less likely to access traditional alcohol treatment services) [18-23]. Although results tend to show small but significant overall effects in favor of Web-based interventions, it is suggested that the public health impact of large-scale usage in a wide range of community settings could be substantial [23].

In addition, Web-based alcohol screening and assessment tools offer health care professionals the ability to rapidly assess their patient’s alcohol use; detect those with potentially harmful patterns of use; and where appropriate, provide them with a BI [24], helping to remove the barrier for clinicians who are reluctant or who feel ill prepared to proactively discuss with patients their alcohol use [6,17,25]. A systematic review conducted in 2014 by Harris et al [26] exploring the efficacy and feasibility of technology-based alcohol screening and BI tools in medical settings found growing evidence of their benefits.

The aim of this study was to develop a novel prototype for a Web-based alcohol risk communication tool for use by primary care health professionals (eg, doctors and nurses) to communicate patients’ risks of alcohol-related harm during routine appointments and to engage with them as to how they can reduce their risk.

### Development

**Figure 1:**
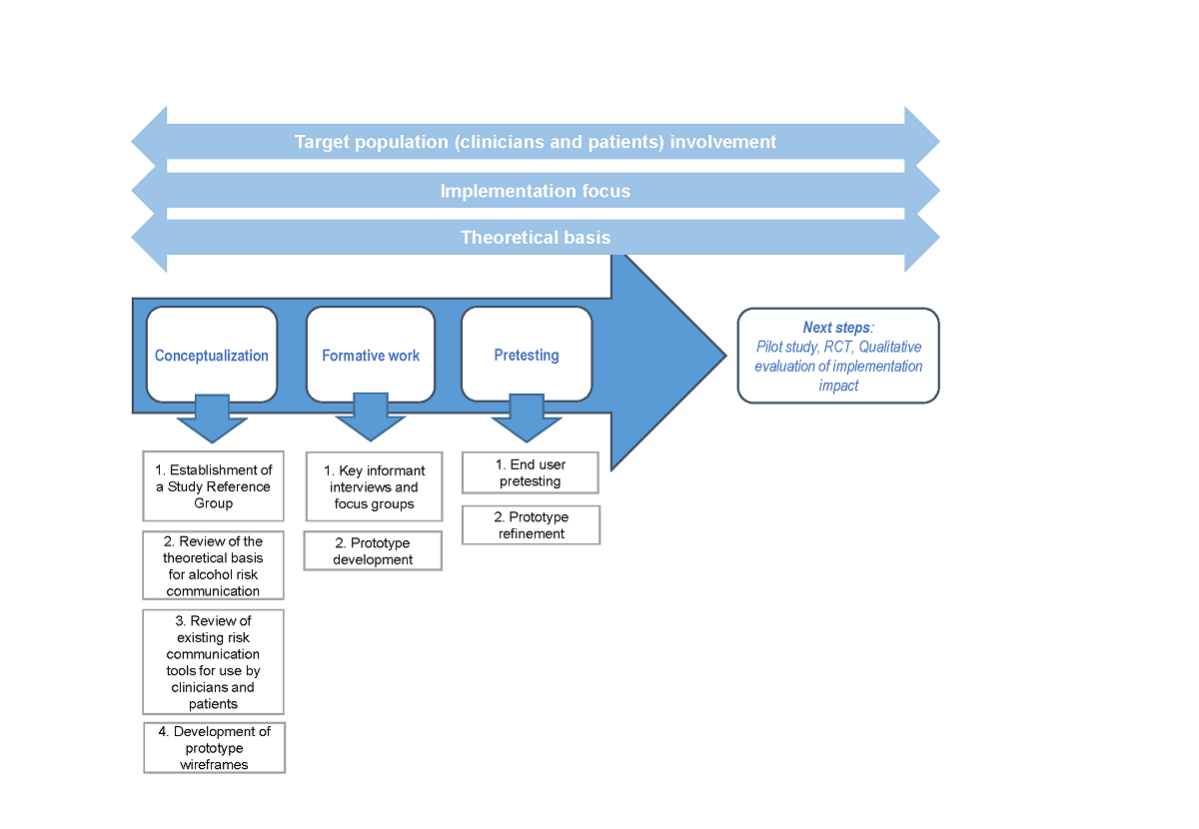
Adapted mHealth Development and Evaluation framework showing stages presented in this paper. RCT: randomized controlled trial.

The development of the alcohol risk communication tool followed the mHealth Development and Evaluation framework [27]. This framework describes a process, using a series of 7 research steps, in which an intervention (or communication approach) is created based on theory and evidence; involvement of the target audience to ensure the intervention is engaging, useful, and culturally appropriate; and a focus on pragmatic implementation from the outset. This approach is robust and has been successfully utilized in a range of mobile health interventions [28-30]. This study has used the following first 3 stages of this framework: conceptualization, formative work, and pretesting (see Figure 1).

#### Conceptualization

An alcohol risk communication tool development team was established to oversee and guide the development process. The team comprised experts in alcohol and injury epidemiology, biostatistics, public health, Māori health, Pacific health, health technology and Web development, health literacy, primary care, risk communication, and drug and alcohol counseling and a health consumer representative. The group provided guidance on all stages of the development and testing of the prototype. During the conceptualization stage, reviews of the relevant evidence and resources were undertaken and discussed with the alcohol risk communication tool development team. Alignment of this information with the stages of change model led to the development of the study’s initial prototype wireframes for the Web-based alcohol risk communication tool.

The initial prototype wireframes were developed for the Web-based alcohol risk communication tool. The purpose of the tool was to provide a framework for use in primary care to communicate alcohol harm risk and the benefit of lifestyle changes. For the tool to communicate risk, it was necessary for the tool to incorporate risk screening. The AUDIT [4] was chosen for this purpose because of it being a commonly used tool in primary health care settings. The initial prototype wireframes include the following screens:

- Screen 1: welcome
- Screens 2 to 4: demographic questions
- Screens 5 to 14: risk screening—the AUDIT alcohol risk screening tool [4] incorporating visual representations of responses to items and pop-up boxes providing further details to assist with completing the screening tool
- Screen 15: current drinking behavior—the AUDIT risk score presented using a risk continuum incorporating traffic light colors to indicate level of risk, interpretation of this in relation to risk, and considerations
- Screen 16: changing drinking behavior—details on where changes can be made to lower risk
- Screen 17: others drinking—a question about concern for others drinking and a pop-up for what signs to look for
- Screen 18 to 19: resources—details of available resources and support and the ability of these to be emailed directly to the patient
- Screen 20: close and thank you

**Figure 2:**
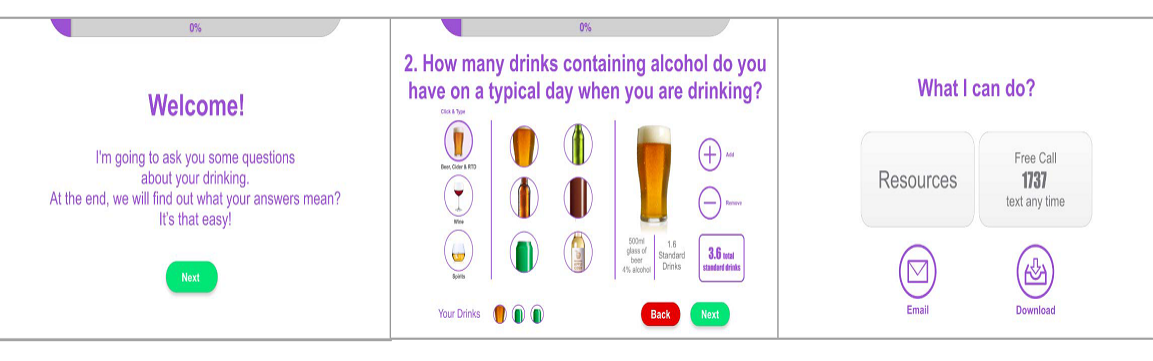
Example screens of the initial prototype.

Example screens from the initial prototype are displayed in Figure 2.

#### Formative Work

Formative work was undertaken to explore ideas about alcohol risk communication, including perceptions about what helps or hinders effective alcohol risk communication in the primary care setting, and to get feedback on the initial prototype. This information was discussed by members of the alcohol risk communication tool development team, and the findings informed changes to the initial prototype, resulting in a revised prototype for pretesting.

#### Pretesting

Pretesting of the prototype by primary care health professionals (eg, doctors and nurses) was undertaken to gain feedback on the tool’s performance in real-world settings as well as its relevance, ease of use, and any suggested refinements.

This paper presents the results of the formative work and pretesting of this tool.

## Methods

### Formative Work

#### Study Design

A mix of focus groups and key informant interviews were conducted with potential end users of the risk communication tool, including primary care health professionals (eg, doctors and nurses) and consumers. We aimed to conduct up to 5 focus groups and 6 key informant interviews.

#### Participants

Participants were identified by the study investigators from existing networks and selected to ensure a range of perspectives representing different health care professionals, representatives of different ethnic groups, and both academic and clinical perspectives. Consumers were identified and invited to participate through the Waitemata District Health Board Community Engagement Team.

#### Procedures

Both interviews and focus groups were semistructured. The domains of inquiry were identified from a review of the relevant published literature and drawing on the expertise of the research team. The domains of interest included the following:

- Perspectives on risk communication
- How risk should be communicated and by who
- Barriers and enablers to risk communication
- Demonstration of the initial prototype using a hypothetical patient
- Look and feel
- Content (eg, language, complexity, and health literacy aspects)
- Functionality
- Acceptability
- Usefulness

All interviews and focus groups were recorded for the purpose of supplementing notes taken. Participants were offered a NZ $20 shopping voucher as an acknowledgment of their participation.

#### Analysis

Inductive content analysis [31] was used to analyze the data obtained from the interviews and focus groups. This approach systematically and objectively reduces the data to concepts that describe the research phenomenon [32]. The results are presented by main category. All data were combined, but where differing views were reported, these were categorized by type: health professional or consumer.

### Pretesting

#### Study Design

Participants completed a Web-based survey followed by a period of pretesting before completion of a follow-up survey. The questions in the Web-based survey were, where possible, drawn from previous relevant research. The questions were piloted with members of the alcohol risk communication tool development team (a number of whom are clinicians), and revisions were made accordingly.

#### Participants

Participants were primary care health professionals (doctors or nurses). Those eligible from the formative work were invited to take part in the pretesting study. Additional participants were identified via existing clinical networks.

#### Procedures

Eligible participants were invited to participate via email. Following consent, participants were emailed a link to a baseline Web-based survey. On completion of the survey, they received access to the Web-based tool. Participants were able to test the tool over a 1-week period before completing a follow-up survey.

#### Measures

Web-based surveys were developed based on a review of surveys used in previous studies of a similar nature. The surveys were built in REDCap software (v8.5.0) and covered the following:

- Baseline survey
- Sample characteristics including demographics and practice characteristics
- Current alcohol screening behavior and barriers to screening
- Perceptions of confidence to identify and manage at-risk drinkers
- Follow-up survey
- Usability and acceptability of the tool
- Suggestions for improvement of the tool
- Perceptions of confidence to identify and manage at-risk drinkers

#### Analysis

Survey data were analyzed and summarized using descriptive quantitative analyses, including means, standard deviation, and proportions. Qualitative comments were analyzed using inductive content analysis. Where relevant, anonymized quotes from participants are used to illustrate key points.

### Ethics Approval

Ethics approval was obtained from the University of Auckland Human Participants Ethics Committee (reference number 020502). Written informed consent was obtained from the focus group, key informant, and pretesting participants before their participation in the studies.

## Results

### Formative Work

A total of 11 key informants and 7 consumers participated in either the focus groups or interviews. There were 2 focus groups completed: the first with 5 health care professionals (4 doctors and 1 nurse) and the second with 7 consumers. A total of 6 individual interviews were completed involving clinicians from Community Alcohol and Drug Services, academics in addiction research, primary care nurses, health literacy experts, consumers, and primary care doctors. Health professionals working directly with Māori and Pacific people participated in focus groups and key informant interviews. The results are presented by the following domains: (1) general risk communication and (2) feedback on the initial prototype.

#### General Risk Communication

Consumers strongly felt that their family doctor was the best person to assess their alcohol risk because of the established relationship and the ability of a family doctor to put the drinking into context, that is, family history, current health, and medications. Similarly, most key informants felt that family doctors are appropriate health professionals to assess alcohol risk. Essential to successful risk communication was that it was personalized, that is, the need to focus on the health effects of drinking in a personalized way, and that it was positively framed and focused on improving outcomes.

The most common barrier to risk assessment and communication identified across all participants was time in the context of competing demands within a consultation. Other barriers identified included the invasive nature of risk assessment, confidence in conducting a risk assessment, personality of the family doctor, embarrassment and denial by patients, perceptions that alcohol risk prevalence is low, and perceptions that the risk and associated harms were not a priority for the patient.

#### Feedback on Initial Prototype

Overall, all key informants and consumers were very positive about the prototype. The participants liked it, felt it was clear and simple, and believed that it would be useful in practice. All participants felt that the tool would be highly acceptable to both clinicians and patients. Key informants identified that the main point of difference with the tool compared with what was already available was that it provided all the pieces in 1 place, that is, the tool included the assessment, its interpretation, ways to make positive changes, and resources, whereas other currently available tools only included 1 or some of these things.

Key informants liked the simplicity of the tool and the use of visual aids and felt that the look was appropriate for the health care setting. Consumers commented that they liked the use of traffic light colors as well as the overall layout and look of the tool. There was feedback from all participants about the length of the tool and the time taken to complete the tool; this related primarily to the AUDIT component of the tool, which was not being evaluated in this project. A potential solution identified by both key informants and consumers was the use of the AUDIT-C initially, with the full AUDIT-10 only being needed for those screened as greater than low risk.

Another common theme was that the tool assumed that the patient was ready to change their behavior and that behavior change had not already occurred. It was, therefore, felt that the tool needed to be refined to take into consideration that changes may already have been made or that the patient may not have prioritized behavior change. It was suggested that the tool could ask about changes in behavior that had already been made and assess motivation to change, which would allow for the identification of discrepancy between current behavior and where they want to be.

Although all participants felt the tool was culturally appropriate, key informants discussed the potential for cultural tailoring and translation of the tool into other languages. Feedback also suggested that tailoring could be expanded beyond culture to other demographic variables, for example, for teenage drinkers. Participants reported the health literacy level to be largely appropriate and that the use of visual aids, that is, risk continuum, helped this.

All key informants highlighted the importance of the tool being embedded and integrated within current patient management systems (ie, linked to the *dashboard* or pop-up within patient record). This was considered vital for getting doctors to use the tool, which, in turn, would be a key factor in the tool’s success.

There were differing views on how the tool should best be administered. Some of the clinicians felt that the tool was appropriate for patient-led administration and that there was opportunity for it to be administered in waiting rooms on a tablet, through kiosks in pharmacies, or to be self-administered outside the clinical setting. Others, including all the consumers, felt that administration of the tool was best led by a clinician. They felt that part of the success of the tool would be because of its use being led by a trusted health care professional. They also expressed concern that not everyone that might benefit from using the tool would have access to the technology to use it. They also stressed that if the tool was used outside the clinical environment, then there needed to be appropriate avenues for follow-up if needed.

All key informants felt that the tool complemented other screening tools used in routine consultations, that is, initial visits and annual checkups. By combining the tool with other screening or processes, they felt it would easily become part of routine care. Key informants did not feel that there would be a need for clinician training to use the tool, but there may be a need for training on how it is delivered. It was recommended that simple supporting documents were made available, such as a small card with an overview of the process.

There were many suggestions for how the tool could be further improved within the interviews and focus groups. In addition to the changes described previously, other specific suggestions for improvement included the following: the addition of information on the specific health effects of alcohol consumption and population norms; the addition of an algorithm on the behavior change slide that shows the patient what they should be focusing on to reduce their risk, that is, if the greatest factor in their risk is the quantity they consume on an occasion, then this should be the one that they are encouraged to work on changing first; the ability to send a text message with the links to resources rather than email; and specify that the suggested resources including the phone and SMS helpline numbers were free to access.

### Refinement of the Tool Based on the Formative Work

On the basis of findings from the formative work, the following changes were made to the prototype:

- The use of the AUDIT-C first to triage low-level drinkers. After completion of the AUDIT-C, if low risk, the patient is then sent directly to the risk summary page, whereas others continue with the full AUDIT-10 assessment
- The addition of a screen for recent or planned changes in drinking behavior
- Clarifying that the resources and services recommended were free for patients to access
- The removal of the email functionality
- The addition of a slider on the behavior change slide, which indicates where the patient is currently in relation to how often they drink, how much they drink, and how often they drink a lot. The patient can move the slider up and down to show how their behavior change in that area will impact on their risk.

### Pretesting

#### Sample Characteristics

A total of 7 people completed the pretesting study, of which 5 were primary care doctors and 2 were nurses. Of the 7 participants, 3 had been in their current role for 5 to 9 years and 4 had been in their current role for 10 years or more. When asked to describe the ethnic makeup of their practice population, 2 participants were predominately European, 1 predominantly Pacific, 1 predominantly Māori, and the remaining 2 were mixed ethnicity. More than half (4/7) of the patient populations were urban low- to mid-socioeconomic status.

#### Current Screening Behavior

At baseline, more than half (4/7) of the participants reported that they *always* screen their patients for alcohol use, with the remainder reporting only screening *sometimes*. When asked about barriers to screening for alcohol use, the most common themes were time constraints (n=5) and the nature of the topic (eg, it being difficult to discuss or offensive, privacy, or patients not being honest; n=5). Other common themes included a lack of access to appropriate skills, resources, or referral options if an issue was identified (n=4) or it not being a priority in the consultation (n=3). Only 1 participant reported a lack of training being a barrier to screening.

#### Perceptions of Confidence to Identify and Manage At-Risk Drinkers

There was a modest increase in confidence to identify and manage at-risk drinkers from baseline to post pretesting (Table 1).

**Table 1:** Participants’ rankings of confidence to identify and manage at-risk drinkers from 1 (not very confident) to 10 (extremely confident), N=7.

Confidence to identify and manage “at risk” drinkers	Baseline, mean (SD)	Follow-up, mean (SD)
Confidence in the ability to differentiate between patients who are “at-risk” drinkers versus those with alcohol use disorders	6.43 (1.99)	7.29 (1.60)
Confidence in knowledge regarding what advice to give at various drinking risk levels	6.57 (2.30)	7.57 (1.72)

#### Usability and Acceptability of the Tool

All 7 participants reported that the tool provided a useful framework for intervention and that if the tool were to become freely available, they would be likely to use it in their practice. There were no reports of technical issues while pretesting the tool.

When asked how acceptable they thought the tool was or would be for their patients, the majority (5/7) reported it would be very acceptable, 2 participants were ambivalent, and none felt it was unacceptable. Participants rated the tools’ ease of use, on average, 8.43 (SD 1.13) on a scale from 1 (extremely difficult) to 10 (extremely easy). Participants were asked whether they thought the tool might be more acceptable by some groups than others; 3 participants reported that it might be more acceptable to younger and more technologically savvy patients, and 1 participant each felt that it would be more acceptable to patients with English as the first language, risky drinkers (over those with established drinking problems), and specific ethnic groups:

Easier for those with English as a first language. Younger or more IT savvy will be more familiar with the format. I think overall it will be pretty acceptable to all.

Participant 02

Older people who are not computer literate may need guidance.

Participant 03

When asked whether they felt the tool had the potential to increase their own confidence in assessing alcohol risks and providing advice to patients, most (5/7) agreed and the remainder stated that it was not applicable as they already had high confidence. Reasons provided for why it increased confidence included it being a simple and straightforward tool (n=2), feeling that it increased the credibility of the clinician (n=1), that it reinforces harm minimization (n=1), and that the use of a visual tool was more engaging for patients (n=1):

I think it will increase the credibility of the clinician. Patients believe this sort of tool - they see the tool telling them something not the person [who might be biased].

Participant 02

To have a visual tool to use with a patient can be more engaging than just hearing advice.

Participant 06

Participants were also asked about whether there were any potential barriers to using the tool in general practice. The most common responses included if it was not integrated into the patient management system or dashboard (n=3), patient literacy skills or English language ability (n=2), limited consultation time (n=2), and that they were already using something else for this purpose (n=1).

#### Suggestions for Improvement of the Tool

The most common reported suggestion for improvement made by participants was to change or remove the pop-up instructions for the questions regarding the quantity of alcohol consumed on a typical drinking occasion (slide 6), which they felt was annoying and unnecessary (n=5). Other suggestions included minor changes to the terminology used on slides, the addition of a tab providing the health care professional with more information about what classifies a patient as low or high risk and associated interventions, and the ability for the data from the tool to be both written into clinical notes and printed in a summary form for the patient to take away.

### Refinement of the Tool Based on the Pretesting

On the basis of the findings and recommendations from the pretesting study, the following changes were prioritized for the final prototype of the tool:

- Removal of the pop-up on slide 6 and placement of simpler instructions on the actual tool rather than via pop-up
- Changes to the terminology on the age screen
- A print summary option that provides a brief summary that the patient can take away, including their risk score, and potential behavior changes, including a free text area to include any personalized actions discussed and available resources
- Minor layout and appearance changes.

**Figure 3:**
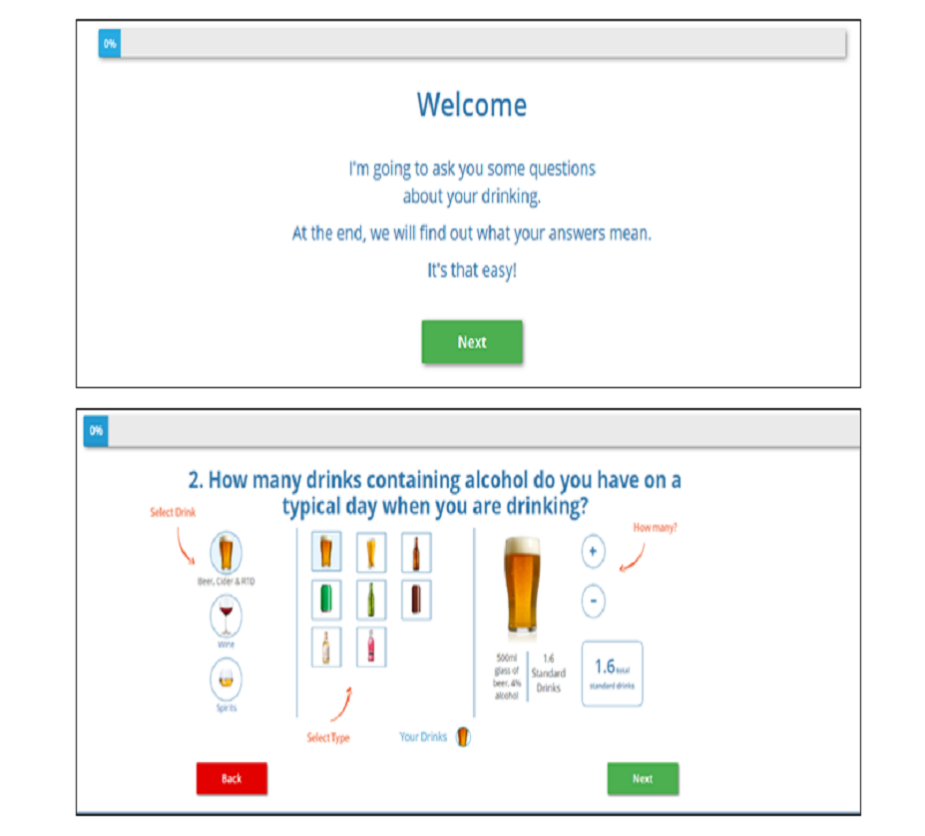
Example screens from the final prototype.

Example screens from the final prototype are displayed in Figure 3. See Multimedia Appendix 1 for the full tool.

## Discussion

### Principal Findings

This study presents the development of a Web-based *alcohol risk communication tool* for use by health professionals in primary health care to communicate alcohol harm risk and the benefit of lifestyle changes to their patients during the course of routine appointments. The conceptualization, formative work, and pretesting of the tool are discussed. The tool was found to be acceptable by primary care health professionals during pretesting, with support for the tool to be made available in practice.

The alcohol screening and BI developed in this study provide a simple but evidence-based tool to screen, present risk, and provide BI to address this risk in a simple, accessible, and user-friendly way. This work builds on the previous evidence supporting the use of alcohol screening and BIs [8,12,13] and Web-based tools for providing more accessible and cost-effective intervention [19].

In line with other published research, our study found that barriers of time, access to resources, and the nature of the topic still exist in primary care [5-7]. There is potential for this tool to overcome some of the previously identified barriers to the implementation of alcohol screening and BI [6,17]. This tool provides a simple framework for communicating risk and providing BI, is quick and easy to use, and includes all steps in 1 place—screening, BI, and suggested resources and support. Findings from the pretesting showed that the tool has the potential to increase the confidence of health professionals in assessing alcohol risks and providing advice to patients because of it being simple and straightforward, because it increased the credibility of the clinician, and because it was engaging for patients. If the tool was to be integrated into patient management systems, it would have the potential to increase the reach of alcohol BI.

### Limitations

The main limitation of this work was the low number of participants (health providers and consumers) representing key priority populations. This limits the generalizability of the findings, and questions remain about the tool’s acceptability to priority populations. It is recommended that further pretesting is conducted with priority populations before the tool is disseminated. Feedback from participants in the formative work indicated that although they felt the tool would be relevant to a diverse population, there was potential for cultural tailoring. The use of further imagery and Te Reo Māori language was suggested. Furthermore, key informants felt that there was potential for the tool to be used with young people; therefore, further work is needed to assess the acceptability in this group.

Although the tool is designed to overcome barriers associated with alcohol screening in primary care, there were some barriers identified in the formative work, which it is not possible to overcome within the scope of this project. Future work is needed to look at how other barriers such as time can be addressed to ensure the widespread adoption of alcohol screening tools.

### Implications for Policy, Practice, and Future Research

Further research is required to assess the feasibility and acceptability of the revised tool in a range of settings. The use of the tool in young people, priority populations such as Māori and Pacific, and rural populations needs to be explored. In addition, although the tool was found to be acceptable and perceived to be useful for providing alcohol screening and BI, further research needs to be conducted to evaluate the effectiveness of the tool for reducing the harmful consumption of alcohol.

The findings of this study have confirmed the benefits of engaging with primary care health professionals in the development of technology-based tools for use in that setting. Designing and developing tools for this setting alongside health care professionals ensures greater acceptability and potential use of the tool but is an aspect of technology development that is often overlooked. Health care professionals in this study (both in the development team and as participants) demonstrated a willingness to be involved in the development process because they want tools that can support improved patient care in the primary care context of time-limited consultations, diverse patient needs, and their patient management systems.

Feedback from this study has highlighted the importance of the tool being integrated into the patient management systems of primary health care. Further work needs to be conducted to explore the best way for this to be done across the range of existing patient management systems used in New Zealand primary health care. It is envisaged that including alcohol screening and BI as part of service-level measures will be the most effective way to support and embed screening and BI into primary care.

Although upstream activities such as policy-level changes are most effective at addressing alcohol harm (eg, pricing, regulation, and limiting access), there remains a need for alcohol screening and BI to support individuals to make positive behavior change in reducing their risk of alcohol-related harm.

### Conclusions

This study describes the development and pretesting of an alcohol risk communication tool, which has the potential to provide an evidence-based and cost-effective tool for addressing harmful drinking in primary care settings. Next steps will explore the integration into primary care patient management systems, the acceptability in priority populations, and its effectiveness.

## Appendix

Multimedia Appendix 1 Screenshots of the final prototype.

## Abbreviations

AUDIT: Alcohol Use Disorders Identification Test
BI: brief intervention
