# Supplementary figures and images for: A Web-Based Alcohol Risk Communication Tool: Development and Pretesting Study
Source: JMIR Form Res. 2020 Jan 2;4(1):e13224. doi: 10.2196/13224 (PMC6966553; doi:10.2196/13224)

# Multimedia Appendix 1: Screenshots of the final prototype.


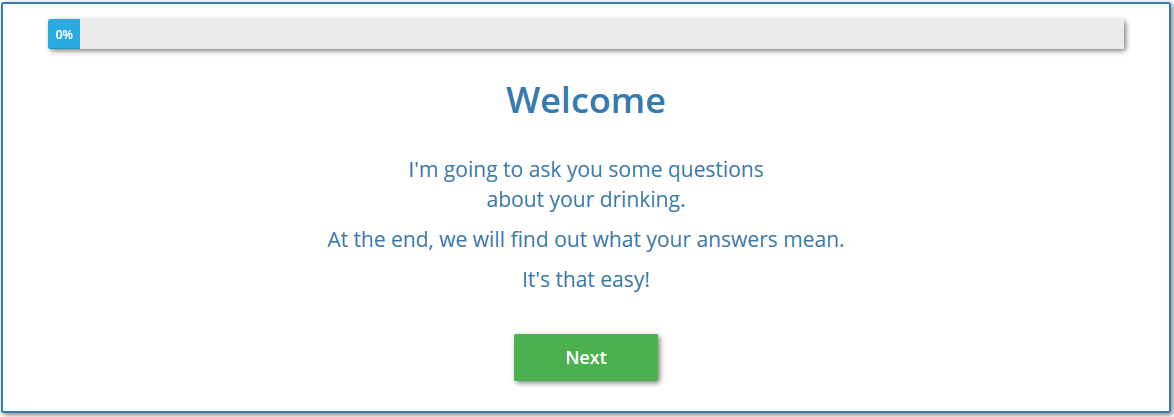


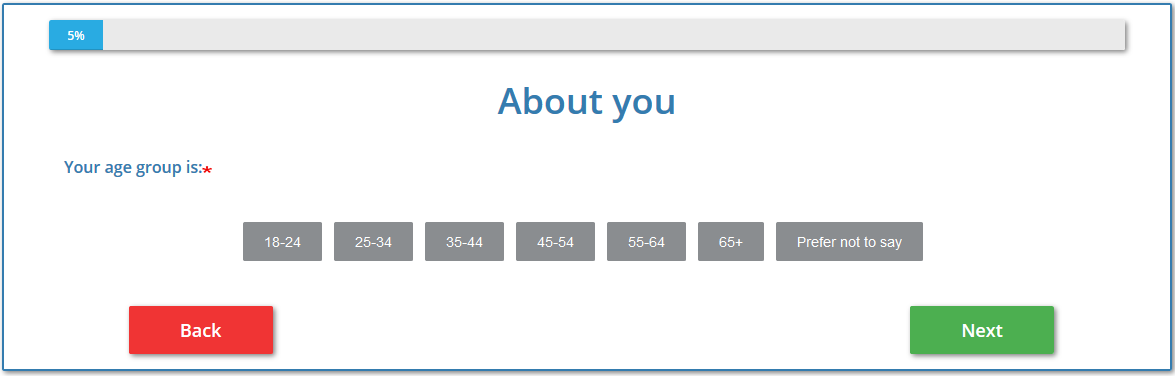


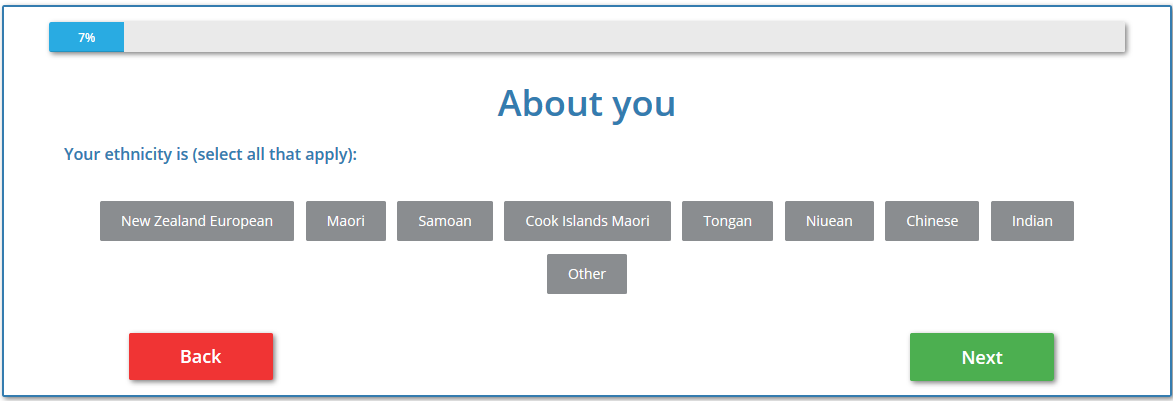


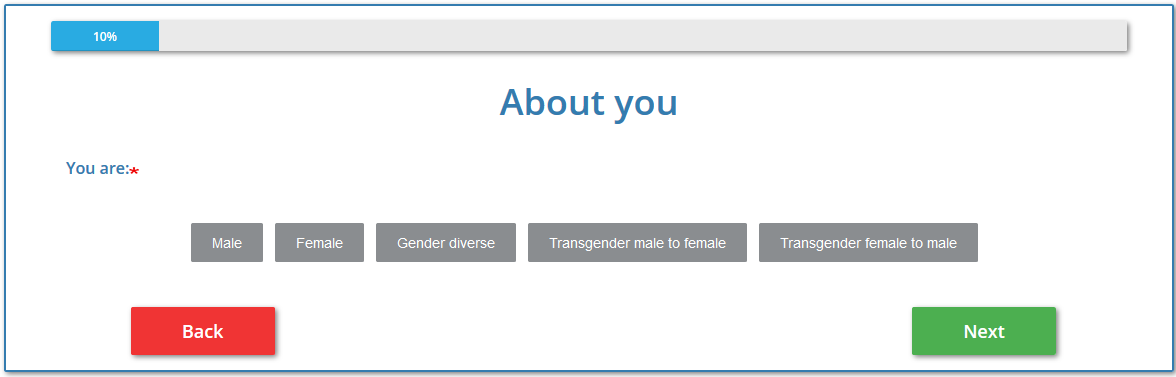


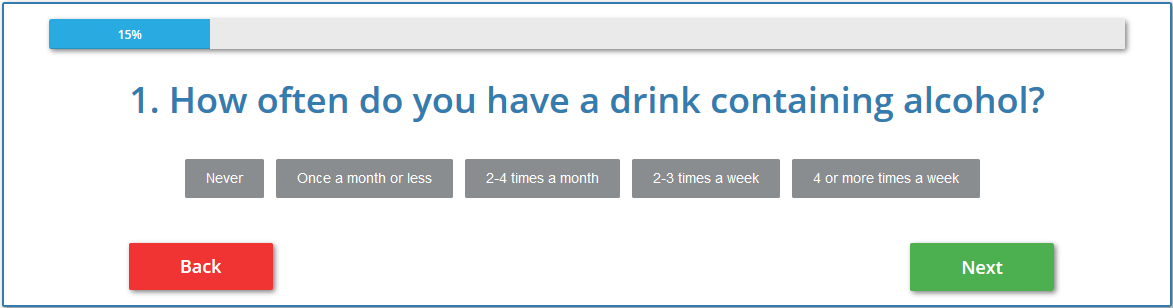


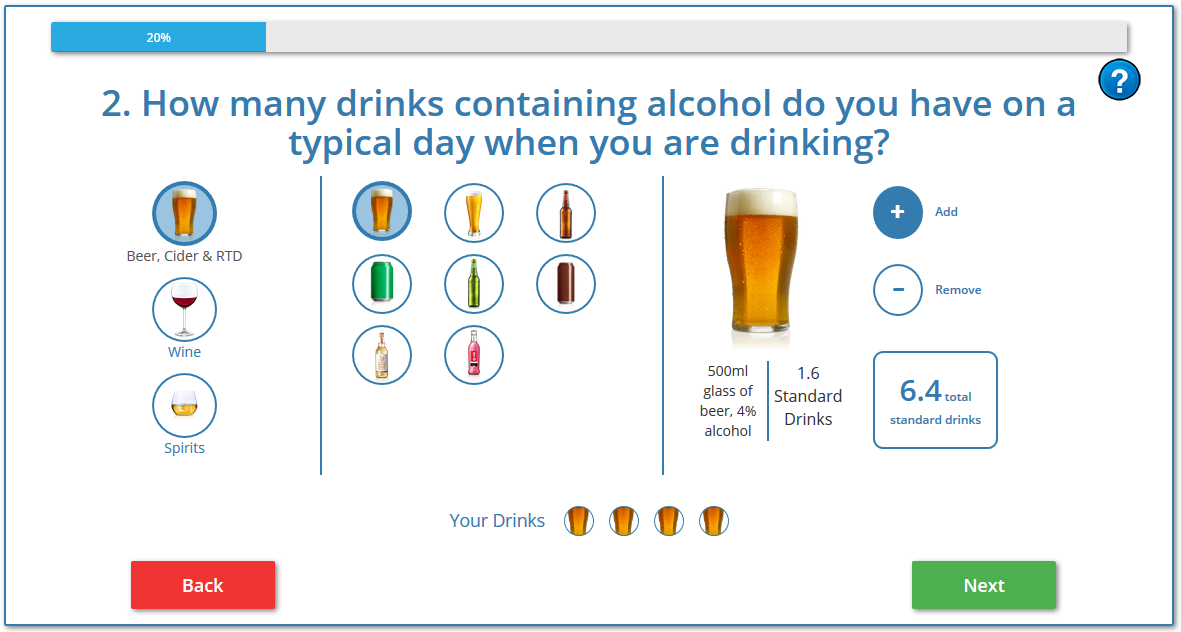


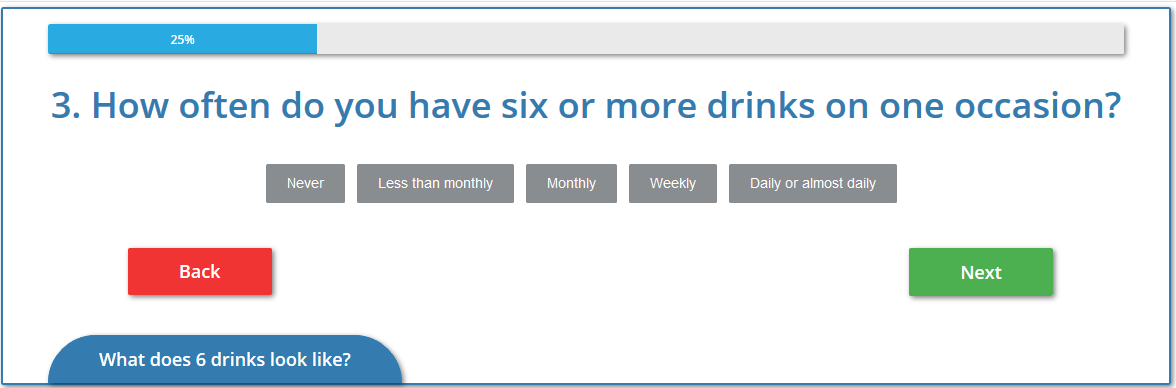


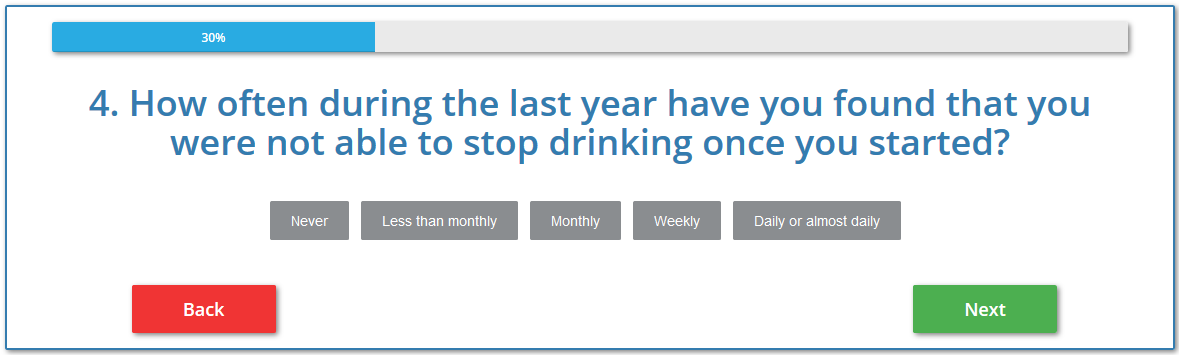


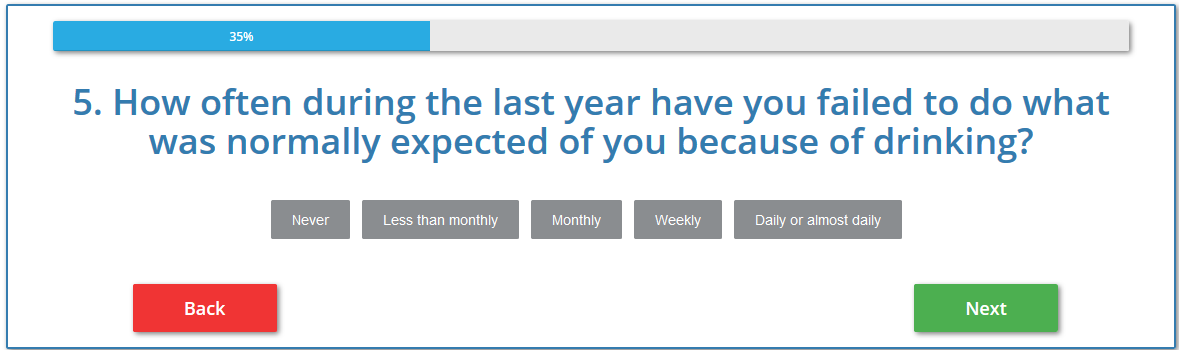


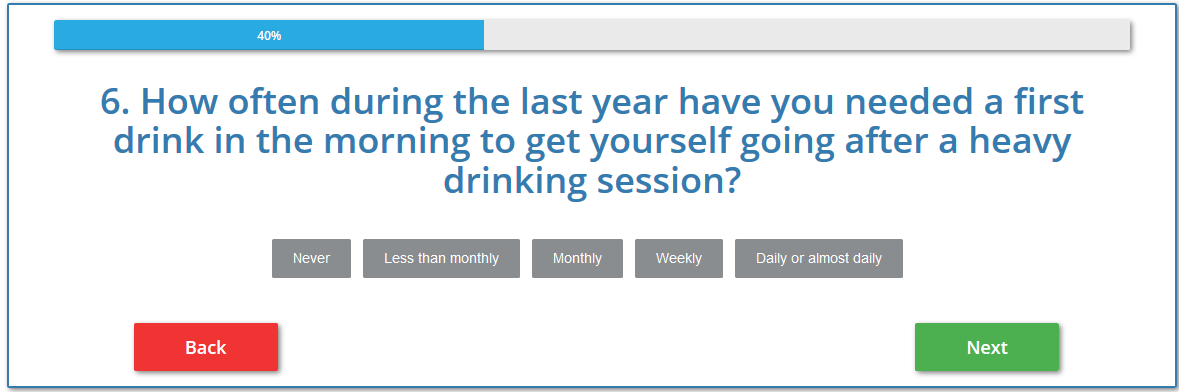


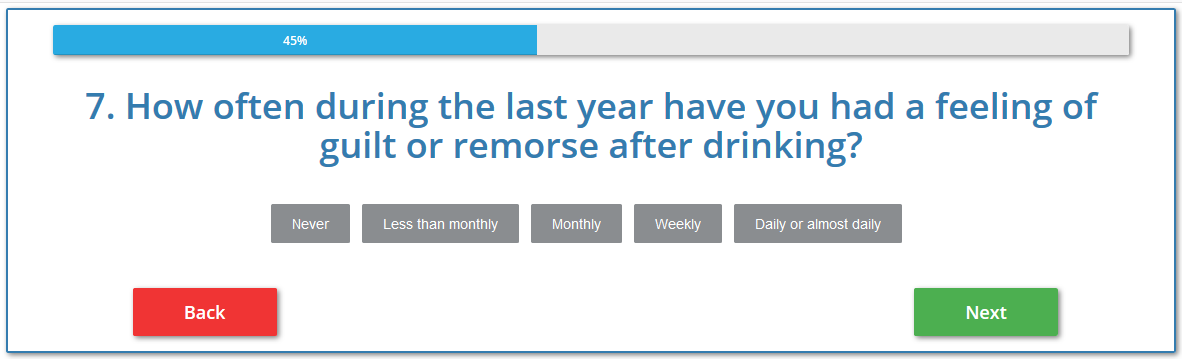


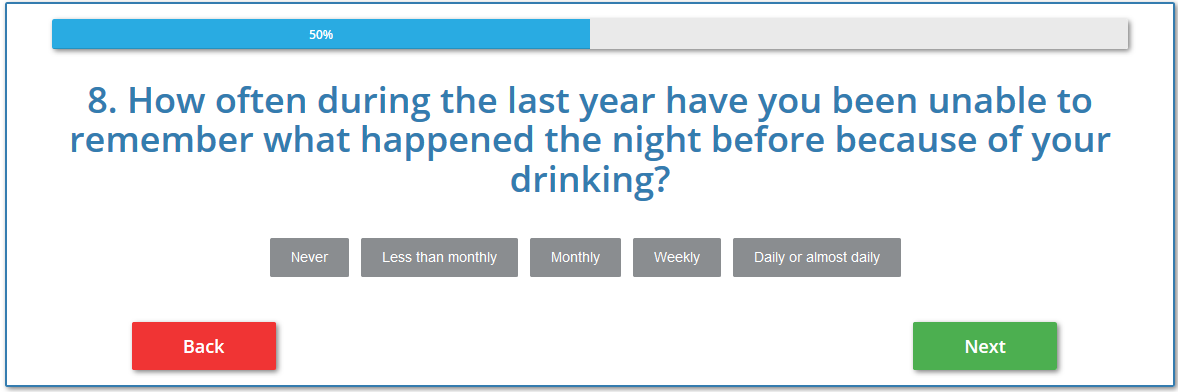


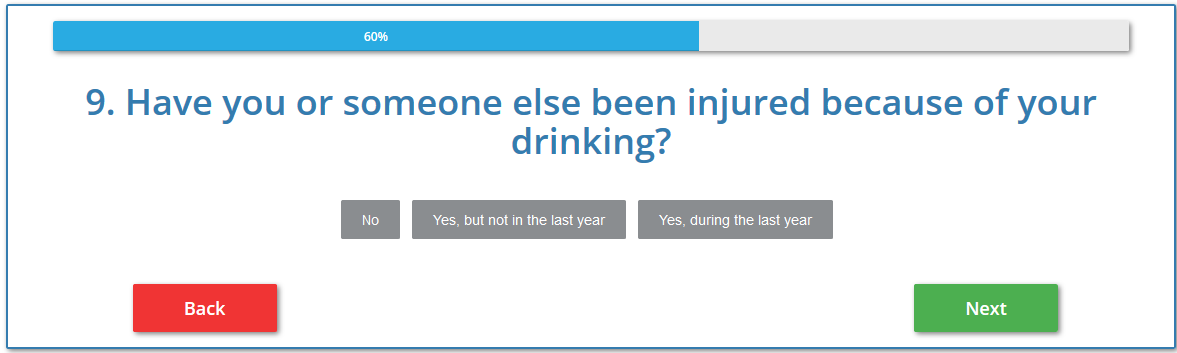


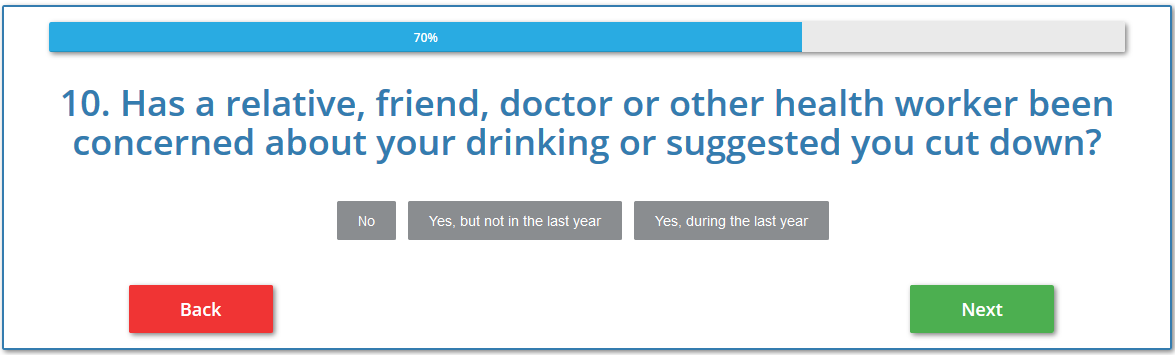


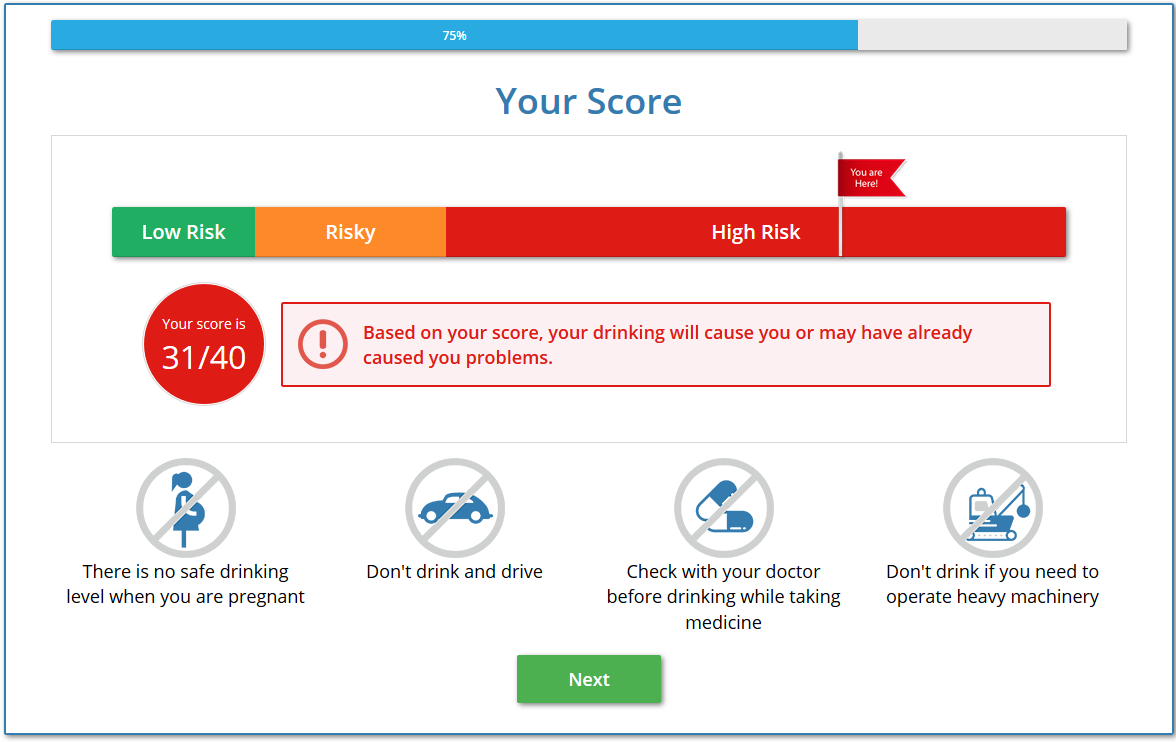


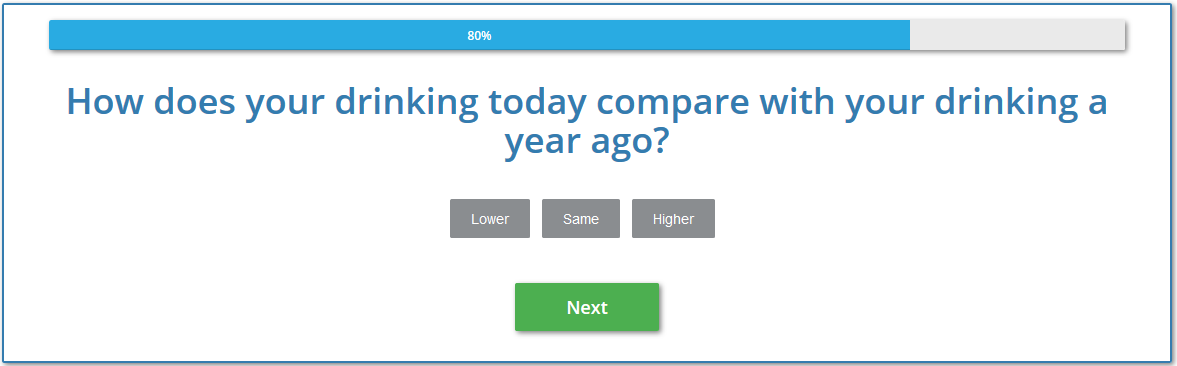


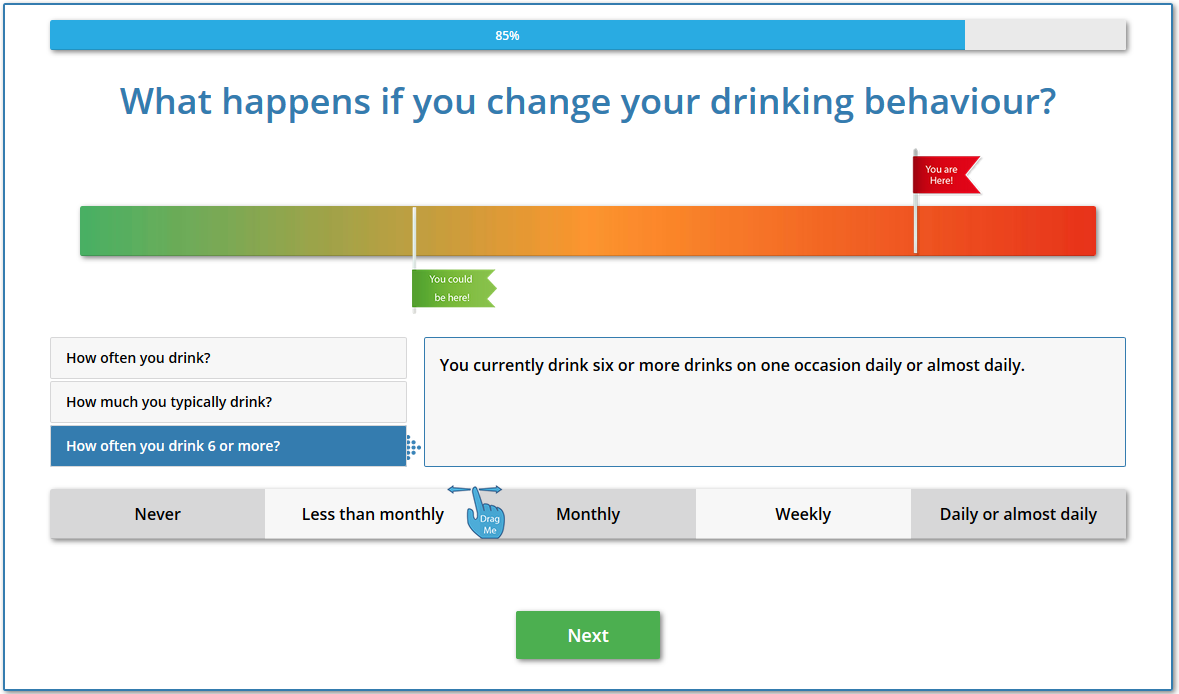


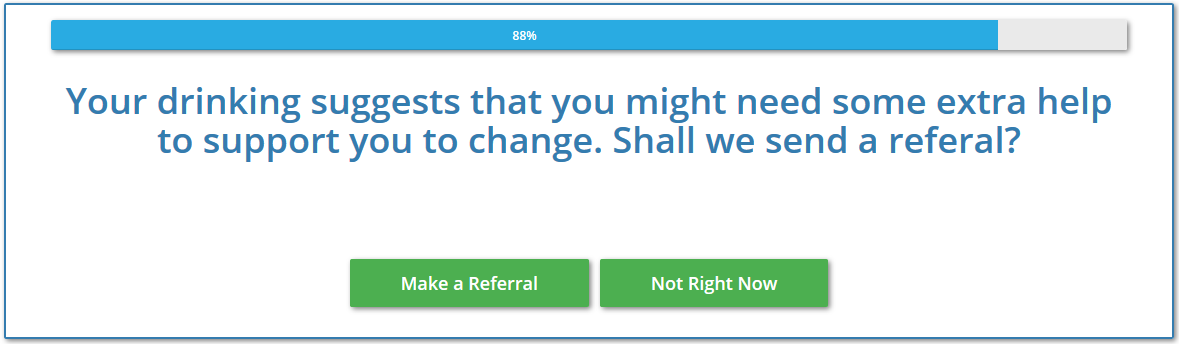


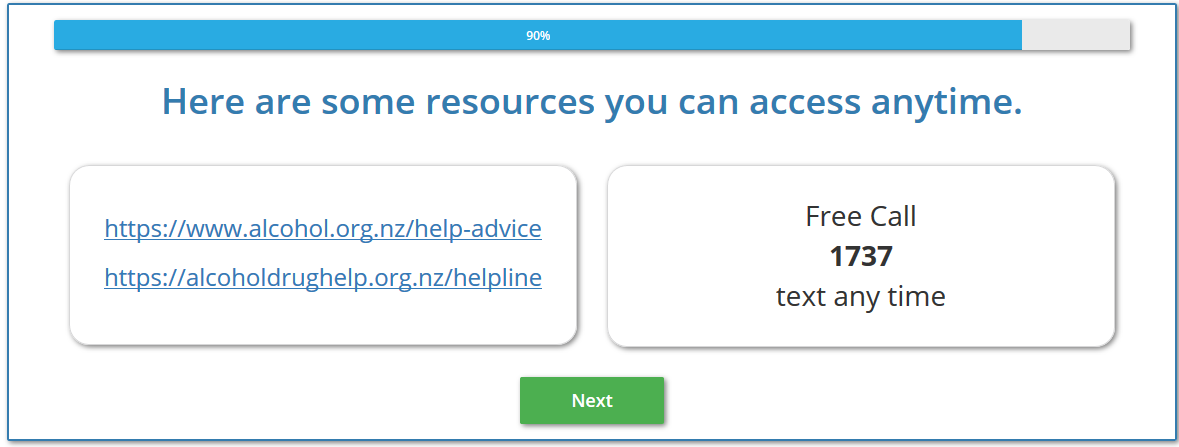


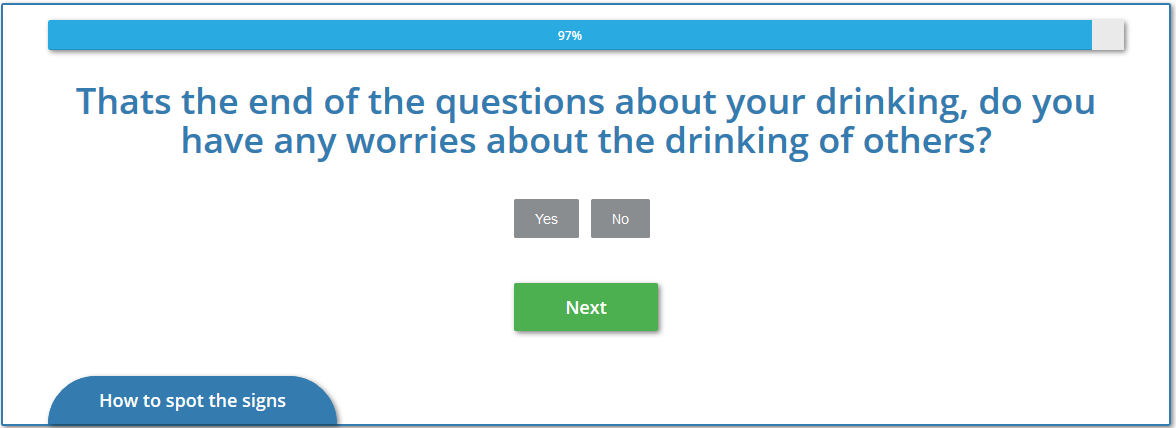


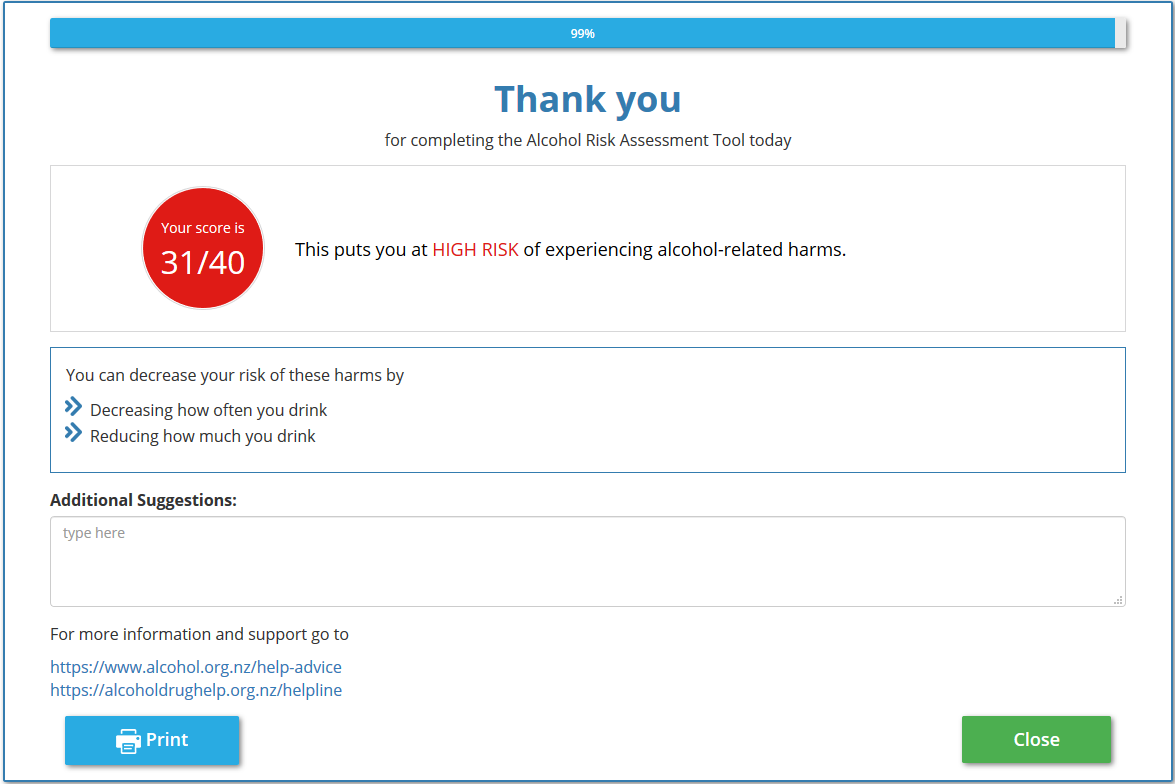


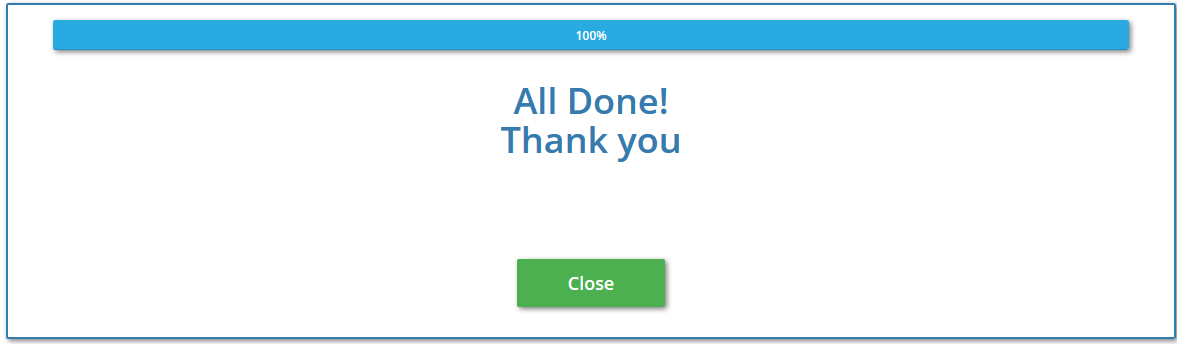

Supplement: Multimedia Appendix 1 [file formative_v4i1e13224_app1.docx]
